# Correlation analysis between morphologic characteristics of the thoracolumbar basivertebral foramen and Kummell’s disease in patients with osteoporosis using imaging techniques

**DOI:** 10.1186/s12891-023-06609-1

**Published:** 2023-06-23

**Authors:** Guang bing Qin, Yi Hua Wu, Huan Shi Chen, Yu Ting Huang, Jun Fei Yi, Ying Xiao

**Affiliations:** 1 Department of Orthopaedic Surgery, Affiliated Liutie Centarl Hospital of GuangXi Medical University, Guangxi Province, Liuzhou, China; 2 Department of Orthopaedic Surgery, Hechi People’s Hospital, Guangxi Province, Hechi, China; 3 Department of Radiological Diagnosis, Affiliated Liutie Centarl Hospital of GuangXi Medical University, Guangxi Province, Liuzhou, China; 4 Department of Orthopaedic Surgery, Affiliated Hospital of Guilin Medical University, Guangxi Province, GuiLin, China

**Keywords:** Basivertebral foramen, Kummell's disease, Senile osteoporosis, Vertebral fissure sign, CT imaging, Correlation analysis

## Abstract

**Background:**

The aging of the population is a social problem faced by many countries in the world. With the increase in the elderly population, the number of patients with Kummell’s disease is also gradually increasing. No study has demonstrated that Kummell’s disease has a clear correlation with the foramen of a vertebrobasilar vein.

**Objectives:**

The research was conducted to describe and evaluate the morphological characteristics of a basivertebral foramen in patients with osteoporosis and Kummell’s disease by CT; to infer whether the specific morphological characteristics of basivertebral foramen may be one of the risk factors of Kummell’s disease; to provide clinical suggestions for the treatment of Kummell’s disease.

**Design:**

Retrospective analysis from January 2020 to December 2021 on 83 patients with 83 vertebral bodies (T8-L5) diagnosed with senile osteoporosis and Kummell’s disease hospitalized in our hospital due to chronic low back pain, including 57 women and 23 men. Group A was assigned for the following patients: the age ranged from 59 to 86 years old, with the average age of 67.30 ± 7.32 years old; the body mass index ranged from 20.01 to 29.46 kg/m^2^, with the average body mass index of 23.51 ± 3.03 kg/m^2^.Group B was assigned for the following patients: 83 patients diagnosed with senile osteoporosis in our outpatient department from January 2020 to December 2021, including 41 males and 42 females; the age ranged from 60 to 85 years, with an average age of 68.52 ± 4.68 years old; the height to weight ratio met the normal reference standard (except 20% above or 10% below the standard weight). Through the lanwon PACS imaging system, the related parameters of the vertebrobasilar foramen in patients with osteoporosis and Kummell’s disease were measured to evaluate and analyze the correlation between the morphological characteristics of the vertebrobasilar foramen in patients with osteoporosis and Kummell’s disease.

**Results:**

In patients with osteoporosis, the distribution of incidence rate of Kummell’s disease in the spine was consistent with that of osteoporotic compression fractures. Sagittal view of the vertebral body on CT scan and the triangular-shaped, trapezoidal-shaped, and irregular-shaped basivertebral foramen in group A accounted for 18%,57%,and 36%,respectively. In group B, triangular-shaped, trapezoidal-shaped, and irregular-shaped foramen accounted for 51%,17%,and 26%,respectively.The distribution of triangular-shaped, trapezoidal-shaped, and irregular-shaped foramen was compared between groups A and B, and the difference was recorded as statistically significant (*P* < 0.05). Additionally, the difference in the distribution of triangular-shaped, trapezoidal-shaped, and irregular-shaped foramen in group A was found statistically significant (*P* < 0.05),while that of Group B was found statistically insignificant (*P* > 0.05).On a horizontal CT scan of the vertebra of group A, triangles, trapezoids, and irregularities accounted for 28%, 26%, and 47%, respectively. In group B, triangles,trapezoids,and irregularities accounted for 31%, 37%, and 30%, respectively. The difference in the distribution of the triangular-shaped and trapezoidal-shaped foramen in groups A and B was statistically insignificant (*P* > 0.05), while that of irregular-shaped was statistically significant (*P* < 0.05). Additionally, there was no statistical significance (*P* > 0.05) in the difference in the morphological distribution of triangular-shaped and trapezoidal-shaped foramen in group A, while that of irregular-shaped was found to be statistically significant (*P* < 0.05). Further, the difference in the morphological distribution of triangular-shaped, trapezoidal-shaped, and irregular-shaped foramen in group B was not statistically significant (*P* > 0.05).In general, about 8% of the vertebral body of BF has an osseous septum. In group A, 97% are single-holed while the remaining 3% are porous; in group B, those with single holes accounted for 76%, while the remaining 24% are porous. In groups A and B, the difference in the morphological distribution of single-holed and multi-holed T8, T11, T12, L1, L2, L4, and L5 vertebral bodies was statistically significant (*P* < 0.05). In group A, the difference in the distribution of single-holed and multi-holed L1 and L5 vertebral bodies was statistically significant (*P* < 0.05). Similarly, the difference in the distribution of single-holed and multi-holed T8, T11, T12, L1, L2, and L4 basivertebral foramen was statistically significant (*P* < 0.05).

**Conclusions:**

In patients with osteoporosis, the incidence of vertebral Kummell’s disease can be associated with the morphological characteristics of the basivertebral foramen, as observed in the CT scan. Furthermore, the vertebral body with trapezoidal-shaped and irregular-shaped basivertebral foramen and boneless septum in the foramen is highly susceptible to Kummell’s disease.

**Supplementary Information:**

The online version contains supplementary material available at 10.1186/s12891-023-06609-1.

## Introduction

Kummell’s disease is a kind of vertebral disease named after six cases of delayed vertebral compression fracture that was first reported by a German doctor named Kummell in 1895 [1]. Most scholars believe that Kummell disease is characterized by multiple degrees of spine injuries, followed by weeks to months of asymptomatic condition before a series of lesions caused by vertebral body injury manifests in delayed vertebral collapse and characteristic intravertebral vacuum cleft (IVC)in imaging examination. Later, the main clinical manifestations include intractable pain in the waist and back, kyphosis, and nerve compression symptoms that may occur in severe cases [2–4]. Kummell’s disease tends to occur in patients with osteoporosis, and according to statistics, about 10% of patients with osteoporosis have vertebral compression fractures (osteoporotic vertebral compression fracture, OVCF). Imaging examinations have revealed that the disease is characterized by vertebral collapse and characteristic vacuum fissure in the vertebral body [5–7].

The aging population is a social problem faced by many countries around the world at present. With the increase in the elderly population, the number of patients with Kummmell's disease has gradually increased [8]. However, there are many theories about its pathogenesis, which are not uniform. In the posterior wall of the thoracic vertebra and lumbar vertebral body and between the two vertebral pedicles, there is an area of bone defects identified as basivertebral foramen (BF), which is the channel of a basivertebral vein (BV), artery, and nerve, in and out of the vertebral body [9–11]. Currently, the research on the particular anatomical structure of BF is only a simple description of its general position. The physical parameters of BF, such as its length, depth, and height, as well as the differences between BF in different segments and individuals, particularly the morphological characteristics of BF in patients with osteoporosis, have not been reported in the literature. Previous studies have verified that the diameter of BF is relatively large in human venous channels, which is important for nourishing vertebral bodies. Additionally, the diameter of BF may be related to vertebral tumor metastasis, infection, bone cement leakage during vertebroplasty, etc. [12, 13]. One of the theories about the pathogenesis of Kummell's disease suggests that the disease is caused by the disorder in blood supply following vertebral body injury, and the basivertebral foramen is the main channel of vertebral nutrient vessels. At present, the correlation between the morphological characteristics of the basivertebral foramen and the pathogenesis of Kummell's disease has not been determined. Therefore, three objectives have been set for this study: (1) to describe and evaluate the morphological characteristics of a vertebral basal foramen in patients with osteoporosis and patients with osteoporosis and Kummell's disease via CT scanning; (2) to infer whether the specific morphological characteristics of a vertebral foramen in patients with osteoporosis might be one of the risk factors for Kummell's disease; (3) to provide references and suggestions for the clinical treatment of Kummell's disease.

## Materials and methods

### Inclusion criteria

(1) The patient has a history of senile osteoporosis; (2) Diagnosis of Kummell’s disease in the patients has been verified by 64-row CT scans; (3) The patient has apparent thoracic and chronic lumbar pain, with a visual analog scale (VAS) score no more than 4 points; (4) The control group was diagnosed with senile osteoporosis in the outpatient department.

### Exclusion criteria

(1) Patients with pathological fracture or who has Acute low back pain was diagnosed as thoracolumbar compression fracture; (2) Patients with a history of thoracic or lumbar open or internal fixation surgery; (3) Patients with severe multiple injuries, hematological diseases, tumors, spinal infections, secondary osteoporosis, and on long-term oral osteoporosis drugs.

### General information

After the screening of patients based on the inclusion and exclusion criteria, a total of 83 vertebral bodies of 83 patients diagnosed with senile osteoporosis and Kummell's disease who were hospitalized due to thoracic or lumbar pain in our hospital from January 2020 to December 2020 were selected (T8-L5), of which 57 are female, and 23 are male. Patients of the age ranged from 59 to 86 years old, with an average age of 67.3 ± 7.32 years old and a body mass index (BMI) ranging from 20.01 to 29.46 kg/m^2^, with an average of 23.51 ± 3.03) kg/m^2^, were assigned as group A. A total of 83 patients diagnosed with senile osteoporosis from January 2020 to December 2021 in the outpatient department of our hospital were randomly selected, which consisted of 41 males and 42 females. Patients of the age ranged from 60 to 85 years old, with an average of 68.52 ± 4.68 years old. The height/weight ratio meeting the normal reference standard (except for those with a weight that is 20% above or 10% below the standard weight) was assigned as group B. Bone mineral density (BMD), and 64-slice CT scan reconstruction of thoracic and lumbar vertebrae were performed in all patients who had met the inclusion criteria. Each segment of the T8-L5 vertebral body was reconstructed independently in sagittal, coronal, and transverse positions. This study was approved by the ethics committee of the hospital, and all patients agreed to voluntarily participate in this study and signed the informed consent.

### Method of measurement

**Fig. 1:**
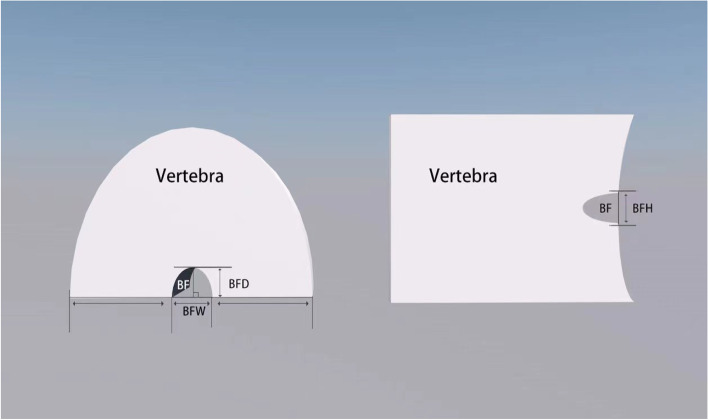
Schematic diagram of basivertebral foramen

The vertebral body-related indexes of patients who had met the inclusion conditions were measured via the CT scan. The measurement parameters include basivertebral foramen width (BFW), and the width of left and right transverse diameter of the same vertebral body (VW), of left and right transverse diameter of the same vertebral body, BF depth (BFD), and the length of anteroposterior diameter of the same vertebral body (VD), of front and rear diameter of the same vertebral body, BF height (BFH) and the height between upper and lower endplates of the same vertebral body (VH) (Fig. 1). The measurement was carried out using a tool provided by the Lanwon PACS Imaging System, with a measurement accuracy of 0. 01 mm. To minimize the experimental error caused by the measurement, the parameters were measured thrice independently by the author, a senior radiologist, and a senior chiropractor, and the average value was calculated. Before conducting the investigation, three surveyors had unified the measurement scheme, the measurement sequence of each index, and the determination of measurement points. For a few vertebrae, it may be difficult to determine the boundary of BF due to the small BF, or bone interference around BF, or the quality of imaging data. At this time, the measurement scheme shall be determined by the three measurers after consultation. Additionally, considering that different individuals and vertebral body sizes may have an impact on the physical parameters of the BF, the relative physical parameters of the BF vertebral body were also calculated in addition to the values obtained by direct measurement. This includes BFW relative value (basivertebral foramen width relative value, BFWr), i.e., BFWr = BFW/VW, BFD relative value (basivertebral foramen depth relative value, BFDr), i.e., BFDr = BFD/VD, BFH relative value (basivertebral foramen height relative value, BFHr), i.e., BFHr = BFH/VH.

### Statistical methods

SPSS 19.0 software was used for statistical analysis. If the measurement data obeyed the normal distribution, the mean ± standard deviation (x ± s) was applied, and the t-test was used for comparison purposes; if the data did not obey the normal distribution, the interquartile spacing of m (P25, p75) was applied, and the rank-sum test was used for comparison purposes. The data count was described by n (%), which was used for the comparison in the χ ^2^ test or Fisher exact probability method. The difference was considered statistically significant when *P* < 0.05.

## Results

**Fig. 2:**
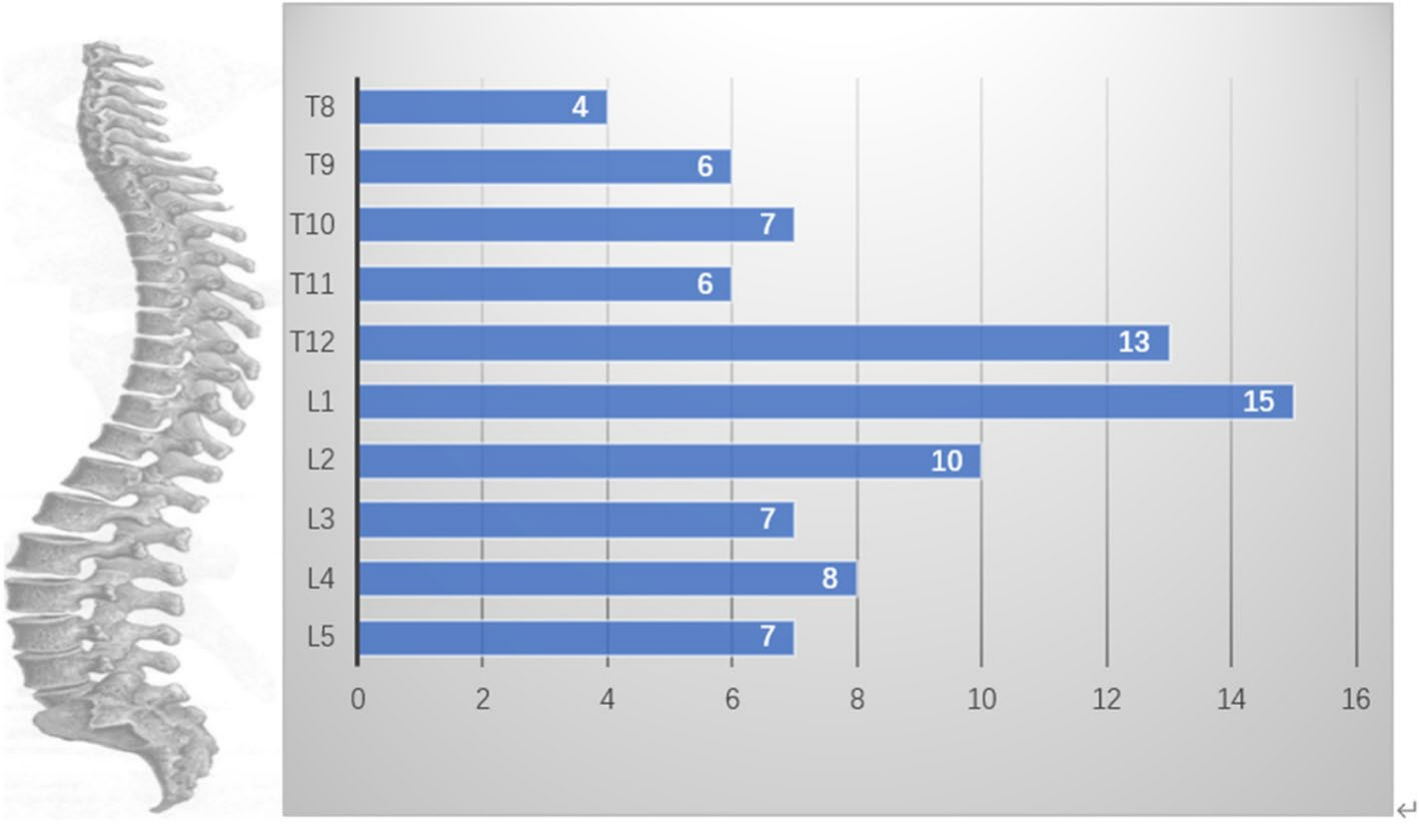
The distribution of diseased vertebrae on the spine (T8-L5) in 83 patients with osteoporosis and Kummell's disease

There was no statistically significant difference (*P* > 0.05) between the two groups, A and B, in terms of sex, age, height-to-weight ratio, and bone mineral density (BMD) (Table 1). Based on the distribution of Kummell's disease in 10 groups (T8-L5) of the spine in 83 patients (Fig. 2), group B was divided into 10 groups (T8-L5), in which the number of vertebral bodies in each group was the same as that in group A.

**Table 1:** Factors that may affect the measurement of parameters of a basivertebral foramen in groups A and B

Factor	group A	group B	t/x^2^	*P*
	（*n*=83）	（*n*=83）		
Gender [*n*（%）]				
Male	26（31.3）	41（49.4)		
			0.42	0.638
Female	57（68.7）	42（50.6）		
Age (years)	67.30±7.32	68.52±4.68	0.726	0.173
Body mass index（BMI）	23.51±3.03	23.01±1.17	9.681	0.747
Bone mineral density T value	-3.71±1.03	-4.02±0.81	1.977	0.079

Analysis of factors that may affect the measurement of parameters of basivertebral foramen demonstrations that there was no significant difference between the two groups in terms of gender, age, height, body mass index, and bone mineral density t-value (*P* > 0.05) (Table 1).

The sagittal position of the vertebral body is visualized by CT scanning. The triangular-shaped, trapezoidal-shaped, and irregular-shaped foramen accounted for 18%, 57%, and 36%, respectively, in group A. The triangular-shaped, trapezoidal-shaped, and irregular-shaped foramen accounted for 51%, 17%, and 26%, respectively, in group B. The comparison of triangular shape distribution, trapezoidal shape distribution and irregular shape distribution between groups A and B was statistically significant (*P* < 0.05). In group A, the difference in the morphological distribution of triangular, trapezoidal, and irregular foramen was statistically significant (*P* < 0.05); In group B, there was no statistical significance in the comparison of triangular, trapezoidal and irregular shape distribution (*P* > 0.05) (Table 2).

**Table 2:** The morphological distribution of basivertebral foramen in sagittal vertebral body of group A and group B (T8-L5)

Vertebral	T8		T9		T10		T 11		T12		L 1		L2		L3		L4		L5	
Group	A	B	A	B	A	B	A	B	A	B	A	B	A	B	A	B	A	B	A	B
triangle	1	2	1	4	2	4	2	4	3	7	4	8	3	5	1	4	2	4	2	4
trapezoid	2	1	2	1	3	1	2	1	6	3	7	4	4	3	3	2	3	2	3	2
irregularity	1	1	3	1	2	2	2	1	4	3	4	3	3	2	3	1	3	2	2	1

In the horizontal position of the vertebral body under CT scanning, triangular, trapezoidal, and irregular foramen accounted for 28%, 26%, and 47%, respectively, in group A; triangular, trapezoidal, and irregular foramen accounted for 31%, 37%, and 30%, respectively, in group B. There was no statistically significant difference between groups A and B in terms of morphological distribution of triangular and trapezoidal foramen (*P* > 0.05). However, in group A and group B there were statistically significant in the comparison between irregular groups (*P* < 0.05). In group A, there was no statistically significant difference (*P* > 0.05) in the morphological distribution of triangular and trapezoidal foramen, while the difference in the distribution of irregular foramen was found as statistically significant (*P* < 0.05). In group B, there was no statistical significance in Pairwise comparison between groups, triangle shape distribution, trapezoidal shape distribution and irregular shape distribution (*P* > 0.05) (Table 3).

**Table 3:** The morphological distribution of basivertebral foramen in the horizontal vertebral body of groups A and B (T8-L5)

Vertebral	T8		T9		T10		T11		T12		L1		L2		L3		L4		L5	
Group	A	B	A	B	A	B	A	B	A	B	A	B	A	B	A	B	A	B	A	B
Triangle	1	1	2	3	2	3	1	2	3	4	4	5	2	4	2	2	3	3	1	3
Trapezoid	1	2	1	2	3	2	1	2	2	5	3	6	3	3	2	2	1	2	2	2
Irregularity	2	1	3	1	2	2	4	2	7	4	8	5	5	3	4	3	4	3	4	2

There are 166 vertebrae in groups A and B, and about 8% of the vertebrae have a bony septum in BF. Based on CT scanning, the image of the coronal position of the vertebral body exhibits a dotted bone cortex in the foramen of the vertebral basal vein at the posterior edge of the vertebral body, which is identified as the BF that is divided into multiple compartments by the existing bone tissue. Group A’s single-holed foramen accounts for 97%, while the remaining 3% are porous; Group B’s single-holed foramen accounts for 76%, while the remaining 24% are porous. The difference in the distribution of single-holed and multi-holed T8, T11, T12, L1, L2, L4, and L5 vertebral bodies between groups A and B was statistically significant (*P* < 0.05); the difference in the distribution of single-holed and multi-holed L1 and L5 vertebral bodies in group A was statistically significant (*P* < 0.05); the difference in the distribution of single-holed and multi-holed T8, T11, T12, L1, L2, and L4 vertebrae in group B was statistically significant (*P* < 0.05) (Table 4).

**Table 4:** The morphology of vertebral foramen in a coronal position of a vertebral body in groups A and B (T8-L5)

Vertebral	T8		T9		T10		T11		T12		L1		L2		L3		L4		L5	
Group	A	B	A	B	A	B	A	B	A	B	A	B	A	B	A	B	A	B	A	B
single-port	4	3	6	6	7	7	6	5	13	11	14	13	10	9	7	7	8	7	6	7
multi-port	0	1	0	0	0	0	0	1	0	2	1	2	0	1	0	0	0	1	1	0

The difference in the BFW values of T8-L5 vertebral bodies between groups A and B was statistically significant (*P* < 0.05); In the intra group comparison of group A, the BFW values of T12 and L1 were statistically significant compared with other T8-11 and L2-5 (*P* < 0.05), while T12 and L1 were no statistically significant (*P* > 0.05); In the intra group comparison of group B, the values of BFW, there were statistically significant (*P* < 0.05). The difference in BFD values of T8-L5 vertebral bodies in groups A and B was statistically significant (*P* < 0.05). In group A, T12 and L1 were compared with other T8-11 and L2-5, all of which were significantly different (*P* < 0.05), while a comparison between T12 and L1 demonstrated no significant difference (*P* > 0.05). In group B, T12 and L1 were compared with other T8-11 and L2-5, all of which were significantly different (*P* < 0.05), while a comparison with T12 and L1 recorded no significant difference (*P* > 0.05). Comparing the BFH values of T8-L5 vertebral bodies between groups A and B demonstrated a statistically significant difference (*P* < 0.05). In group A, a comparison of T12 and L1 with other T8-11 and L2-5 showed a statistically significant difference (*P* < 0.05), while no statistically significant difference (*P* > 0.05) was recorded in comparison with T12 and L1. All pairwise comparisons in group B demonstrated a statistically significant difference (*P* < 0.05)(Table 5).

**Table 5:** Absolute values of parameters of a basivertebral foramen in groups A and B as viewed by CT scanning(*n* = Number of vertebral segments $\overline{x}\pm \mathrm{s }$)

	BFW		BFD		BFH	
	Group A	Group B	Group A	Group B	Group A	Group B
T8	3.16 ± 1.21	3.71 ± 1.37	4.33 ± 1.16	3.71 ± 1.09	3.74 ± 1.62	4.21 ± 1.48
T9	3.41 ± 1.15	3.64 ± 1.13	5.46 ± 0.81	4.50 ± 0.88	3.62 ± 1.27	4.18 ± 0.65
T10	3.74 ± 1.25	4.01 ± 1.71	4.91 ± 1.79	3.11 ± 1.46	4.09 ± 1.57	4.81 ± 1.60
T11	4.11 ± 1.64	4.96 ± 2.05	5.62 ± 0.73	4.20 ± 1.39	4.29 ± 0.70	5.08 ± 0.97
T12	4.83 ± 1.06	5.22 ± 1.70	7.31 ± 1.15	6.71 ± 1.42	5.71 ± 1.74	6.16 ± 1.73
L1	4.59 ± 2.02	7.01 ± 1.16	6.91 ± 1.64	6.33 ± 0.72	6.37 ± 1.29	6.24 ± 0.91
L2	5.41 ± 1.65	5.75 ± 1.38	6.11 ± 1.51	7.01 ± 0.62	7.41 ± 1.25	6.33 ± 1.20
L3	6.82 ± 1.76	7.21 ± 1.73	7.38 ± 1.47	6.81 ± 1.66	6.97 ± 1.78	7.62 ± 1.19
L4	7.01 ± 1.55	7.24 ± 1.07	6.84 ± 0.72	5.91 ± 0.27	6.69 ± 1.52	7.01 ± 1.57
L5	7.41 ± 1.22	7.81 ± 1.75	7.36 ± 1.68	6.79 ± 1.66	7.08 ± 1.77	7.37 ± 1.41

Comparing the BFWr values of T8-L5 vertebral bodies between groups A and B demonstrated no statistically significant difference (*P* > 0.05). Similarly, all pairwise comparisons in groups A and B revealed no statistically significant difference (*P* > 0.05). In addition, a comparison of the BFDr values of T8-L5 vertebral bodies between groups A and B demonstrated statistically significant difference (*P* < 0.05). Similarly, all pairwise comparisons in groups A and B recorded no statistically significant difference (*P* > 0.05). Comparing the BFHr values of T8-L5 vertebral bodies between groups A and B depicted statistically significant difference (*P* < 0.05). Likewise, all pairwise comparisons in groups A and B demonstrated no statistically significant difference (*P* > 0.05)(Table 6).

**Table 6:** Relative values of basivertebral foramen parameters in groups A and B (*n* = Number of vertebral segments $\overline{x}\pm \mathrm{s }$)

	BFWr		BFDr		BFHr	
	Group A	Group B	Group A	Group B	Group A	Group B
T8	0.197 ± 0.021	0.204 ± 0.018	0.312 ± 0.027	0.206 ± 0.015	0.147 ± 0.137	0.210 ± 0.036
T9	0.210 ± 0.015	0.202 ± 0.073	0.247 ± 0.025	0.205 ± 0.013	0.152 ± 0.079	0.197 ± 0.070
T10	0.205 ± 0.020	0.185 ± 0.035	0.346 ± 0.019	0.198 ± 0.026	0.174 ± 0.014	0.215 ± 0.131
T11	0.211 ± 0.019	0.197 ± 0.023	0.220 ± 0.048	0.201 ± 0.021	0.139 ± 0.072	0.202 ± 0.021
T12	0.187 ± 0.013	0.201 ± 0.013	0.310 ± 0.094	0.183 ± 0.003	0.157 ± 0.031	0.211 ± 0.019
L1	0.200 ± 0.037	0.196 ± 0.041	0.292 ± 0.069	0.205 ± 0.071	0.161 ± 0.017	0.224 ± 0.019
L2	0.191 ± 0.024	0.214 ± 0.019	0.328 ± 0.015	0.207 ± 0.025	0.143 ± 0.059	0.213 ± 0.135
L3	0.220 ± 0.033	0.182 ± 0.061	0.281 ± 0.055	0.197 ± 0.062	0.158 ± 0.121	0.190 ± 0.081
L4	0.224 ± 0.074	0.217 ± 0.047	0.274 ± 0.051	0.212 ± 0.077	0.161 ± 0.055	0.224 ± 0.135
L5	0.217 ± 0.068	0.221 ± 0.039	0.309 ± 0.027	0.237 ± 0.020	0.149 ± 0.172	0.197 ± 0.089

**Fig. 3:**
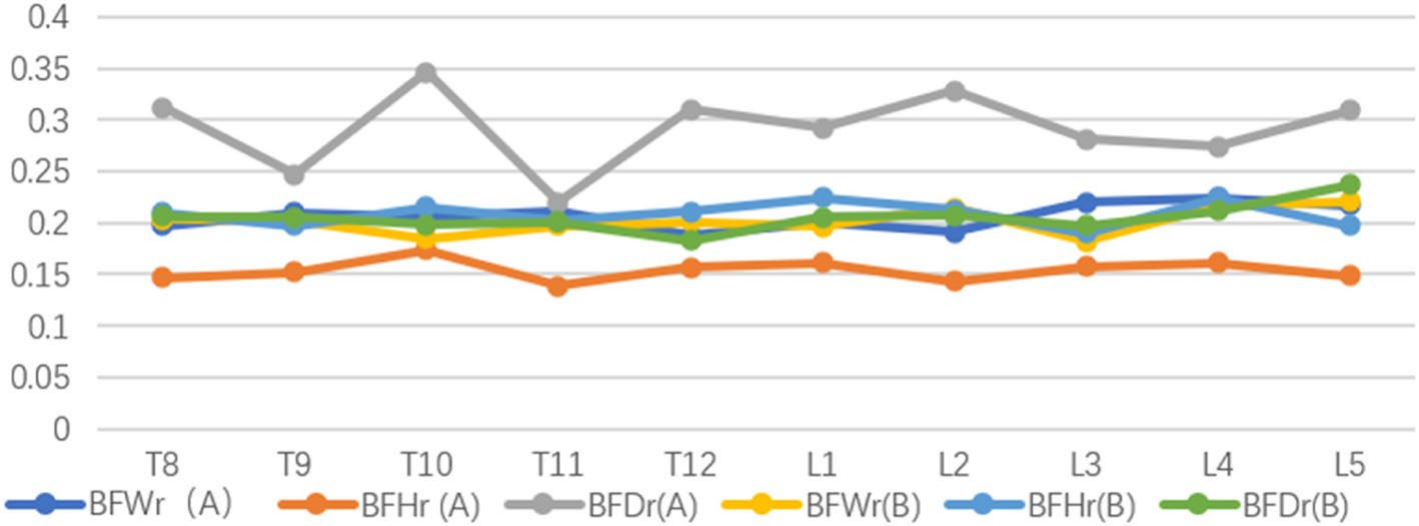
The trend in the mean values of basivertebral foramen parameters of T8-L5 vertebral bodies in groups A and B

We found that the maximum width, depth, and height of BF in groups A and B were close to or more than 1/4 of the width, depth, and height of the same plane of the measured vertebral body. The average width, depth, and height were about 1/5 of the width, depth, and height of the same plane of the vertebral body. In group A, the BFDr was higher than that of other groups, and the relative value of BFHr was lower than that of other groups (Fig. 3).

## Discussion

In this study, a total of 166 vertebral bodies (T8-L5) (Fig. 2) in patients with osteoporosis and osteoporosis complicated with Kummell's disease were scanned by 64-sliced CT, and the physical parameters of vertebral basivertebral foramen were measured with lanwon PACS software(Fig. 1). After excluding interference factors such as gender, age, BMI, and Bone mineral density T value (Table 1). It was found that the morphological characteristics of a basivertebral foramen in a patient with osteoporosis and osteoporosis complicated with Kummell's disease were not identical.

### Anatomical features of a basivertebral foramen in patients with osteoporosis

There are few anatomical descriptions of a basivertebral foramen in professional anatomical textbooks. An anatomical observation by Ma C M et al. was made on 23 pairs of dry adult lumbar vertebrae, revealing that the bone density around the horizontal and sagittal sections of lumbar BF was similar to that of the surface of the vertebral body. It was also discovered that the surrounding bone was loose, and bone canals surrounded by bone trabeculae connected with BF at the coronal level. About 6% of the BF has a bony septum, divided into 1–8 compartments, as observed in the ordinal thoracic and lumbar vertebrae [14, 15]. It has been reported that with the increase in the number of the vertebral body, the height and width of the vertebral body from thoracic vertebra to lumbar vertebra also gradually increase, and the bone spacing in BF gradually transits from horizontal arrangement to vertical distribution. In this study, the characteristics of a basivertebral foramen in patients with osteoporosis were found to conform to these changes [16, 17]. This study found that about 8% of patients with osteoporosis have bone septum in the foramen of the basivertebral (Table 4). The images revealed that the BF morphology of each vertebral body is different in both sagittal and horizontal positions, which can be classified into triangular-shaped, trapezoidal-shaped, and irregular-shaped foramen. The CT scanning images of the vertebral bodies in a sagittal position of group B reveal that the triangular-shaped, trapezoidal-shaped, and irregular-shaped foramen account for 51%, 17%, and 26%, respectively, while in a horizontal position, they account for 31%, 37%, and 30%, respectively (Tables 2–3). In terms of the BF parameters of group B, it was observed that the proportion of BFH in each measured vertebral body was smaller than that of BFW. Considering the different degrees of compression changes in the vertebral bodies of patients with osteoporosis, the maximum width, depth, and height of BF are close to or more than about 1/4 of the width, depth, and height of the same plane of the measured vertebral body, in addition, the average width, depth, and height are about 1/5 of the width, depth, and height of the same plane of the vertebral body (Fig. 3). It was verified that the diameter of BF is significantly larger than that of the nutrient vessel pores in the bones of other parts of the body, such as the femur, etc. The large diameter of BF, in addition to the routine drainage of blood inside the vertebral body, allows for proper adjustment of the volume of venous blood entering and leaving the vertebral body, as well the volume of the vertebral body and the metabolic activity of the bone in the vertebral body [13, 18–21]. A histological study found that the bone trabeculae forming the BF hole wall are a horizontal plate-shaped structure, which mainly bears the longitudinal load. BF has been identified as a weak area when bearing the longitudinal load because there is no supporting bone in the middle of the hole, unlike the bone trabeculae in other parts of the vertebral body that are cylindrical and parallel to the longitudinal axis of the vertebral body. Thus, the larger the diameter of the hole, the weaker the BF in terms of ability to bear the longitudinal load [22]. In addition, because the bone is brittle, when the stress is applied to the vertebral body, the stress is concentrated around the BF, particularly at the tip and in the posterior half of the vertebral body resulting in vertebral fracture. The fracture line is easy to pass through the BF, which has also been verified by the profile that illustrates the correlation between the basivertebral foramen and the development of Kummell's disease [23]. O’Connor SD [11] et al. studied the causes of fracture blocks at the posterior edge of the vertebral body by using the vertebral fracture model, which suggested that the vertebral fractures were related to the morphology of BF. From the perspective of biomechanics, the lack of stress-bearing capacity of BF as a channel may be a potential risk factor for thoracic and lumbar vertebral compression fractures and the formation of posterior vertebral edge fractured blocks. Similarly, in patients with osteoporosis, different forms of BF also undergo different degrees of changes upon exposure to stress.

### Anatomical characteristics of a basivertebral foramen in osteoporosis complicated with Kummell's disease

Maldague et al. first proposed that the intravertebral vacuum cleft (IVC) is related to Kummell's disease in 1978. Later, many scholars reported that IVC was identified through the X-ray examination patients with Kummell's disease [24–27]. IVC is caused by the accumulation of gas in the vertebral body, typically located in the middle of the vertebral body or near the side of the compressed vertebral endplate. IVC is more apparent in the anterior part of the vertebral body than in the posterior part and most often involves the thoracolumbar and lumbar joint (T12-L1), particularly the T12 [28, 29]. This study found that in patients with osteoporosis and patients with osteoporosis complicated with Kummell's disease, the maximum width, depth, and height of BF are close to or more than about 1/4 of the width, depth, and height of the same plane of the measured vertebral body. In addition, the average width, depth, and height account for about 1/5 of the width, depth, and height of the same plane of the vertebral body (Fig. 3). The BFDr (A), the relative value of the horizontal depth of the vertebrobasilar foramen of group A, was higher than that of group B, whereas the BFHr (A), the relative value of basivertebral foramen height of group A, was lower than that of group B (Tables 5–6). The differences are attributed to the different degrees of changes in the compression of vertebral bodies in patients with osteoporosis. The higher BFD in group A than that of group B is attributed to the conditions of the vertebral body that are associated with Kummell's disease and the apparent vertebral fissure, which might have affected the measurement of BFD. This finding suggests the connection between vertebral fissure and vertebral basal vein foramen. The effects of osteoporotic lesions on the vertebral body are relatively low at its posterior edge, which is mainly manifested in the morphological changes of the basivertebral foramen. However, such slight change often greatly impacts the blood vessels passing through the basivertebral foramen, which affects the supply and circulation of blood in the vertebral body. By measuring a vertebral basal venous foramen in patients with osteoporosis and Kummell's disease, this study found that the height of basivertebral foramen in patients with Kummell's disease is significantly smaller than that of patients with osteoporosis. However, there is no significant difference in the width of basivertebral foramen between the two groups. Therefore, special attention should be paid to this parameter in the vertebroplasty treatment of Kummell's disease. Although the height of the basivertebral foramen may change, the width of the basivertebral foramen may not change significantly, signifying the potential risk of bone cement leaking into the spinal canal through the basivertebral foramen during operation.

### The pathogenesis of Kummell's disease

For a long time, several orthopedic doctors have considered that Kummell's disease is equivalent to IVC, which mostly occurs in patients with acute or chronic osteoporotic fractures and is one of the main causes of pain due to long-term instability of the vertebral body [30]. Research has found that Kummell’s disease is related to alcohol abuse, diabetes, long-term use of adrenal glucocorticoids, radiation therapy, liver cirrhosis, pancreatitis, vasculitis, type I Gaucher disease, and other factors. These factors may induce the disease by aggravating vertebral ischemic necrosis and hindering the post-traumatic repair of vertebral bodies [31, 32]. Furthermore, Kummell's disease often occurs in patients with osteoporotic vertebral compression fractures, signifying the association of the disease with osteoporosis [33]. Considering these factors, the mechanism of the disease may be associated with the occurrence of microfracture due to osteoporosis, which destroys the morphology of the basivertebral foramen, and damages the arterioles in the vertebral body and the veins passing through the basivertebral foramen, thereby affecting the blood supply and circulation in the vertebral body, the interaction between them further aggravates the disruption of blood supply in the vertebral body, which suggesting osteoporosis as one of the pathogenesis of vertebral ischemic necrosis [34, 35]. According to the literature, several pathological studies on Kummell's disease have demonstrated that the vertebral trabeculae of patients with Kummell's disease are typically characterized by ischemic necrosis, dead bone formation, and formation of cavity fissures without bone tissue. These findings suggest that the occurrence of ischemic bone necrosis is one of the important mechanisms of Kummell's disease [36, 37]. Kim [38] considered that under the conditions of osteoporosis, trauma might lead to the fracture of the cancellous bone of the spine, resulting in the formation of a micro hematoma that hinders the local blood supply. In addition, the bone trabecula may be subjected to ischemic necrosis, leading to the hardening of the broken ends, while the bone trabecula in the central area of necrosis is absorbed, forming a fissure parallel to the endplate. As the stress of the cancellous bone around the fissure grows larger and minor trauma is prone to fracture, and forming the vicious circle of ischemia-fracture-ischemia [2, 39]. Finally, the vacuum sign of the vertebral body parallel to the endplate is formed, and the collapse of the vertebral body signifies the occurrence of Kummell's disease [40].

### Correlation between changes of basivertebral and Kummell's disease

**Fig. 4:**
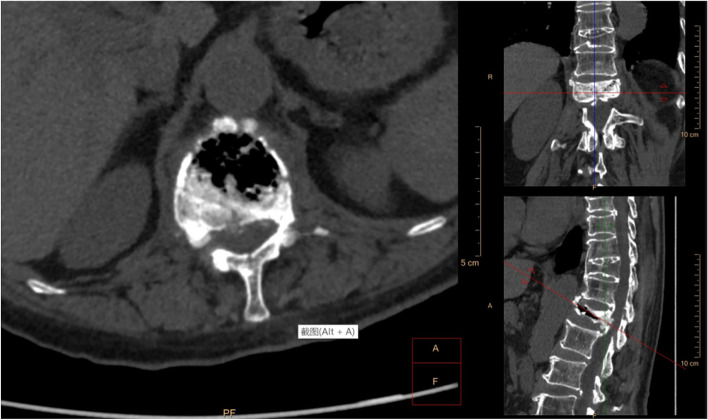
Sagittal, coronal and horizontal three-dimensional CT images of T12 vertebral body in patients with osteoporosis and Kummell's disease

**Fig. 5:**
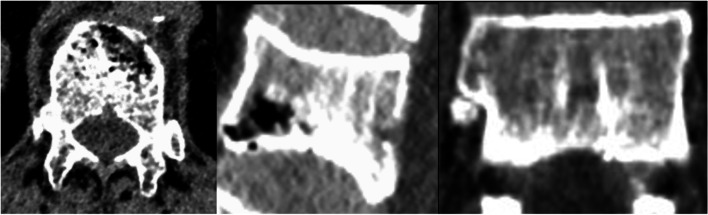
CT scan sagittal, coronal and water shape of vertebroid foramen of T12 vertebral body in patients with osteoporosis complicated with Kummell's disease

**Fig. 6:**
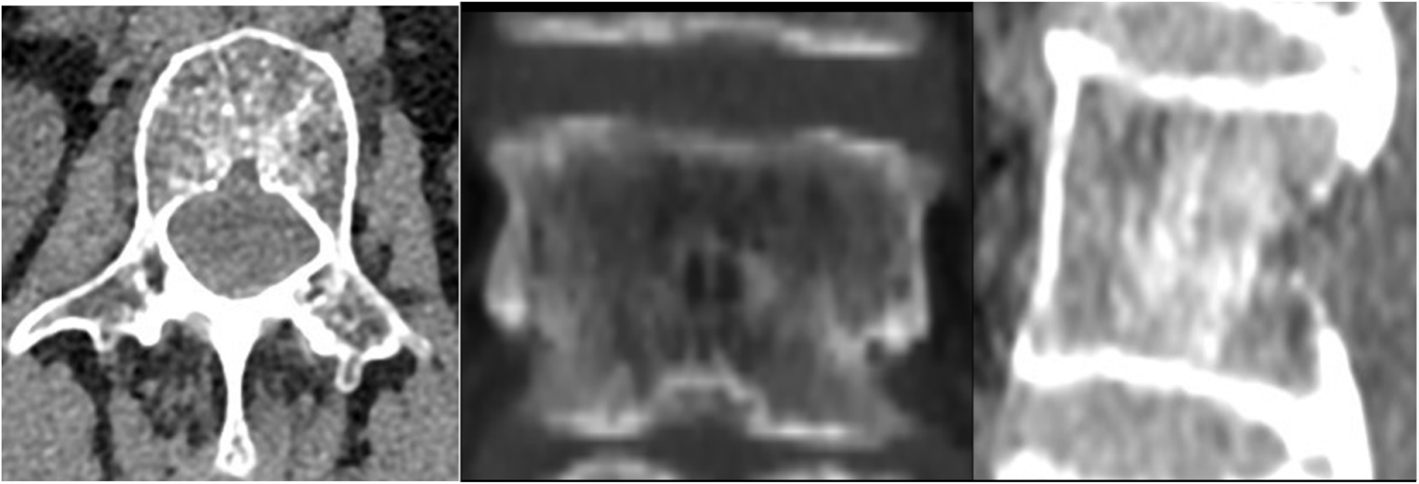
CT scan sagittal, coronal and water shape of vertebroid foramen of T12 vertebral body in patients with osteoporosis

This study found that in patients with osteoporosis and patients with osteoporosis complicated with Kummell's disease, the maximum width, depth, and height of BF demonstrate a certain ratio in the transverse diameter, anterior–posterior diameter and height of the vertebral body at the same level. It is inferred that there is a certain degree of connectivity between the basivertebral foramen and the vertebral fissure formed by Kummell's disease (For example, Figs. 4, 5and 6 Morphological characteristics of the T12 vertebra in osteoporosis combined with Kummell's disease). In addition, it was found that the single-holed trapezoidal and irregular basivertebral foramen are more prone to Kummell's disease (Tables 4, 5 and 6). These changes in the vertebral basal venous foramen impact the blood supply in the vertebral body. Under the conditions of osteoporosis, a cycle of ischemia-fracture-fracture-ischemia in the vertebral body is initiated, which affects the blood circulation in the vertebral body, thus aggravating the expansion of the vertebral fissure, leading to the formation of Kummell’s disease and the expansion of the fissure space. Been et al. suggested that the presence of the basivertebral foramen leads to differences in the biomechanical characteristics of different regions in the vertebral body, which leads to specific changes in the vertebral body upon exposure to external forces. The osteoporotic vertebral body often exhibits the signs of the intravertebral fissure, which is associated with Kummell’s disease [41].When the vertebral body is damaged, and compression fractures occur at varying degrees, the original bone trabeculae are destroyed, and the pores in the vertebral body are interconnected to form pores with a larger diameter. Also, the mechanical weakness of the vertebral basal foramen area may lead to serious injury in bone trabeculae, resulting in the collapse of the basivertebral foramen channel and disruption in blood circulation in the vertebral basal vein and lumbar artery branches passing through the channel. Consequently, the ischemia of the vertebral body will be initiated, which may lead to the abnormal bone metabolism associated with the damaged vertebral body and the non-uniform of vertebral fracture [42]. These findings suggest the correlation between the development of Kummell's disease and the basivertebral foramen from the perspective of hemodynamics and biomechanics.

## Conclusions

This study considered that under the conditions of osteoporosis, the morphology of BF in the thoracic and lumbar vertebrae could be characterized by collapses, narrowing, and connection with the vertebral fracture line following the damage to the vertebral body. Consequently, the blood supply in the vertebral body is affected, leading to poor nutrient levels and the destruction of the metabolic microenvironment of the vertebral bone cells, leading to the bone cells' apoptosis, which creates non-uniformed fractures. In addition, under the combined action of stress and other factors, the bone trabeculae at the fracture collapse, and the cavities and fissures continue to expand, further developing into Kummell's disease. Anatomical observation via imaging technique carried out in this study allows for an observation of the location of some BF. While most BF can be visualized by 64-row CT scanning, the smaller bony foramen is difficult to visualize. Despite limitations in the observation method used in this study, the technique used is simple and allows for observation and measurement of large sample sizes.

## Supplementary Information

**Additional file 1.**

## Data Availability

All relevant data are provided in the manuscript are availabled from the corresponding author upon reasonable request.

## Abbreviations

IVC: Intravertebral vacuum cleft
OVCF: Osteoporotic vertebral compression fracture
BF: Basivertebral foramen
BV: Basivertebral vein
VAS: Visual analog scale
BMI: Body mass index
BMD: Bone mineral density
BFW: Basivertebral foramen width
BFD: Basivertebral foramen depth
BFH: Basivertebral foramen height
VW: Width of left and right transverse diameter of the same vertebral body
VD: Length of anteroposterior diameter of the same vertebral body
VH: Height between upper and lower endplates of the same vertebral body
BFWr: Basivertebral foramen width relative value
BFDr: Basivertebral foramen depth relative value
BFHr: Basivertebral foramen height relative value
